# Modulation by luminal factors on the functions and migration of intestinal innate immunity

**DOI:** 10.3389/fimmu.2023.1113467

**Published:** 2023-02-13

**Authors:** Masaaki Higashiyama, Soichiro Miura, Ryota Hokari

**Affiliations:** ^1^ Department of Internal Medicine, National Defense Medical College, Tokorozawa, Japan; ^2^ International University of Health and Welfare, Tokyo, Japan

**Keywords:** leukocytes, innate immunity, luminal factors, neuropeptides, migration, gut immunity, inflammatory bowel disease, intestinal allergy

## Abstract

Luminal antigens, nutrients, metabolites from commensal bacteria, bile acids, or neuropeptides influence the function and trafficking of immune cells in the intestine. Among the immune cells in the gut, innate lymphoid cells, including macrophages, neutrophils, dendritic cells, mast cells, and innate lymphoid cells, play an important role for the maintenance of intestinal homeostasis through a rapid immune response to luminal pathogens. These innate cells are influenced by several luminal factors, possibly leading to dysregulated gut immunity and intestinal disorders such as inflammatory bowel disease (IBD), irritable bowel syndrome (IBS), and intestinal allergy. Luminal factors are sensed by distinct neuro-immune cell units, which also have a strong impact on immunoregulation of the gut. Immune cell trafficking from the blood stream through the lymphatic organ to lymphatics, an essential function for immune responses, is also modulated by luminal factors. This mini-review examines knowledge of luminal and neural factors that regulate and modulate response and migration of leukocytes including innate immune cells, some of which are clinically associated with pathological intestinal inflammation.

## Introduction

Luminal antigens, nutrients, metabolites from commensal bacteria, bile acids, or neuropeptides influence immune cell trafficking in the intestine, and for this mechanism in the gut, chemokines, cytokines, and adhesion molecules are involved. In Crohn’s disease (CD), one of the inflammatory bowel diseases (IBDs), nutritional therapy with reduced enteric antigenic load is effective to induce remission (1), suggesting that modulation of luminal factors exert strong effects on functions of immune cells. Immune cell trafficking from the blood stream through the lymphatic organ to the lymphatics has also an essential function for immune responses, and most mature leukocytes spend much of their cellular lives trafficking around the body to identify and eradicate microorganisms and malignant cells (2). This trafficking system works systemically including gut-associated lymphoid tissue (GALT). Aberrant trafficking is deeply involved with pathogenesis of IBD, and, for example, blockade of adhesion molecules such as α4 β7-integrin has been proven to be clinically effective (3). The expression of chemokines and its receptors also regulate intestinal regional immunity depending on the location and inflammation, some of which are therapeutic candidates in IBD (4). For example, the receptors CCR9 and CXCR3 and their respective ligands CCL25 and CXCL10 have received attention (4).

Among the immune cells in the gut, innate immune cells play an important role for the maintenance of intestinal homeostasis through a rapid immune response to luminal pathogens. Macrophages, dendritic cells (DCs), neutrophils, basophils, eosinophils, mast cells, natural killer (NK) cells, and innate lymphoid cells (ILCs) are included in innate immunity. Among them, DCs play an important role in the initiation and regulation of immune responses by recognizing and responding to pathogen- and danger-associated signals (5). Mast cell secretes a diverse array of biologically active compounds including proteases upon some stimulations, leading to allergen-induced inflammation (6). ILCs comprised of ILC1, ILC2, and ILC3, which produce Th1-, Th2-, and Th17-related cytokines, respectively, in response to intestinal stimulation, leading to the stimulation of the immune response and tissue repair (7). Intestinal ILCs have been also reported to travel from the intestinal mucosa to the mesenteric lymph nodes (MLNs) in response to luminal water or intestinal inflammation (8, 9).

**Figure 1:**
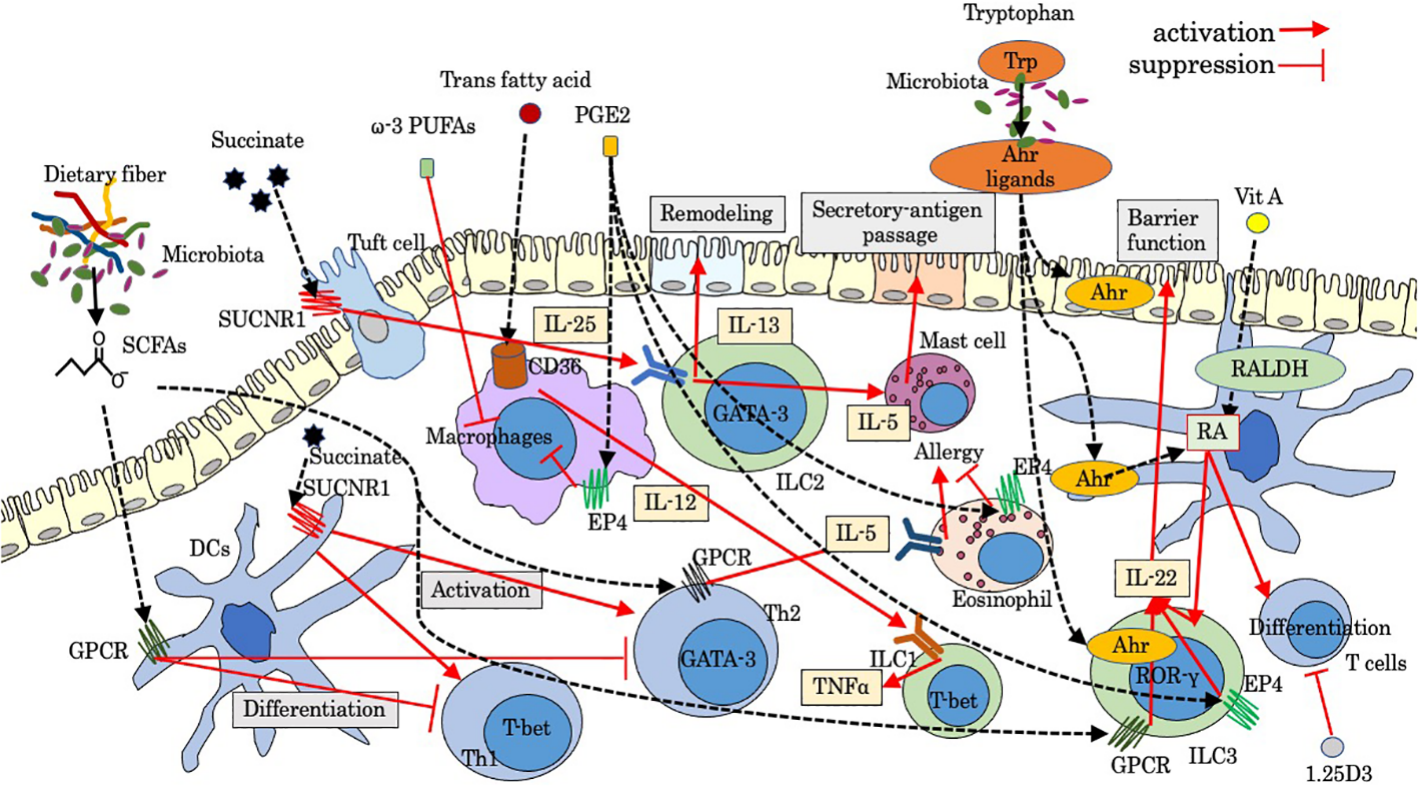
Modulation by luminal factors on intestinal innate immunity. Ahr, aryl hydrocarbon receptor; GPCR, G-protein-coupled receptor; PGE2, prostaglandins E2; PUFAs, poly unsaturated fatty acids; RALDH, retinaldehyde dehydrogenase; RA, retinoic acid; SCFAs, short-chain fatty acids; SUCNR1, succinate receptor; Trp, tryptophan; 1.25D3, 1,25-dihydroxyvitamin D3.

**Figure 2:**
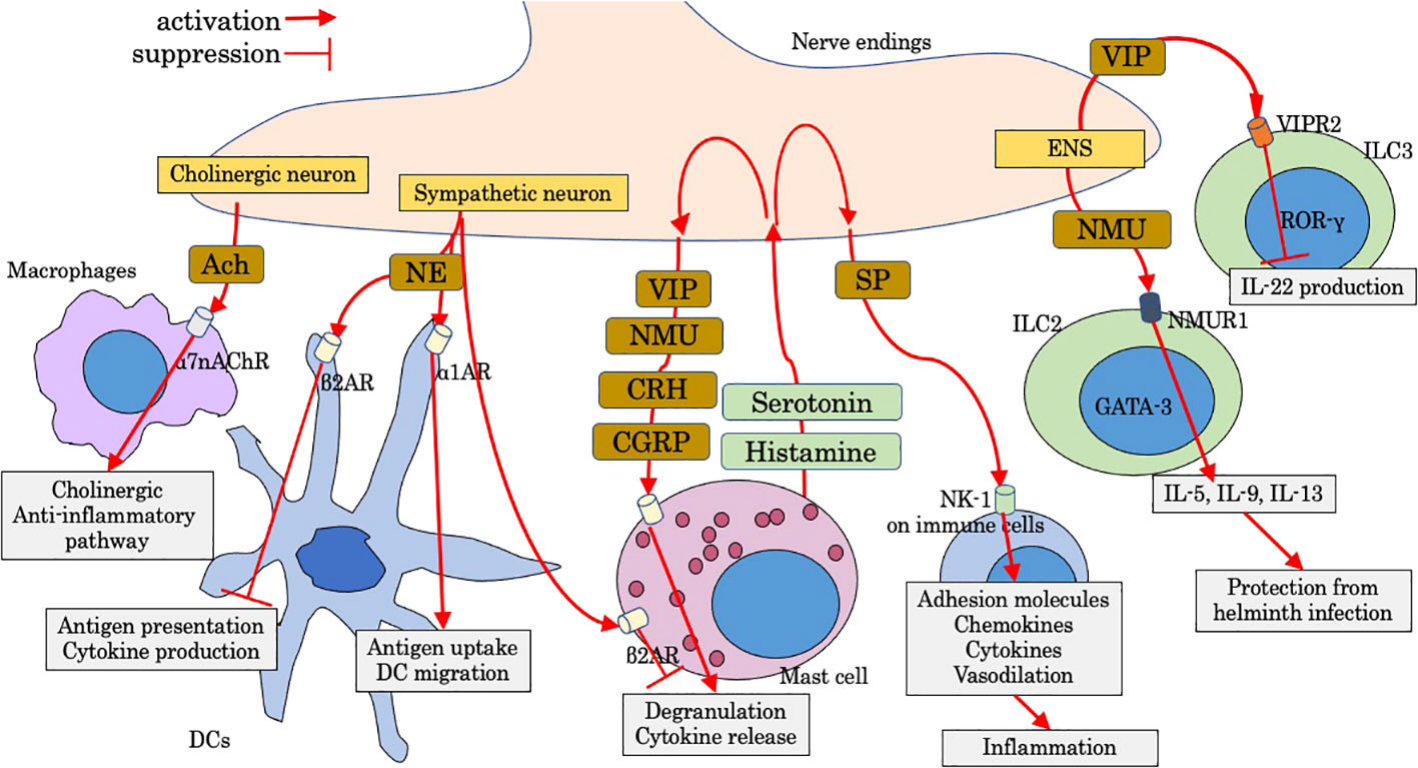
Modulation by neuropeptides on intestinal innate immunity. Ach, acetylcholine; CGRP, calcitonin-gene-related peptide; CRH, corticotropin-releasing hormone; DC, dendritic cell; ENS, enteric nervous system; NE, norepinephrine; NK-1, neurokinin-1; NMU, neuromedin U; NMUR1, Neuromedin U receptor 1; SP, substance P; VIP, vasoactive intestinal peptide; VIPR2, VIP receptor type 2; α1AR, α1-adrenergic receptor; α7nAChr, α7 nicotinic acetylcholine receptor; β2AR, β2-adrenergic receptor.

Luminal factors, such as diet ingredients and derivatives and microbiota and its metabolites, are crucial regulatory factors for host immunity, some of which could lead to dysregulated gut immunity and intestinal disorders such as IBD and intestinal allergy. Microbiome modulation can be a new therapeutic approach for gut immunity, the beneficial effects of which are dependent on their metabolisms and metabolic by-products such as short-chain fatty acids (SCFAs) (10). Tryptophan metabolism under the influence of microbiota is also pivotal for gut immunity (11). Luminal factors are also sensed by distinct neuro-immune cell units, which also have a strong impact on the immunoregulation of the gut. Recent studies have gradually revealed the effect of luminal and neural factors on gut immunity including innate immune cells. In this mini-review, we aimed to examine as much as knowledge of luminal and neural factors that regulate response and migration of leukocytes with main focus on innate immune cells, some of which are involved in pathological intestinal immunity. Figures show schematic summaries of modulation by representative luminal factors (**Figure 1**) and neuropeptides (**Figure 2**) on intestinal innate immunity introduced in this manuscript.

### Dietary fiber metabolites

#### Short chain fatty acids

Relatively high levels of SCFAs including acetate, propionate, and butyrate and other fatty acids are produced by anaerobic bacteria, such as Firmicutes, as a metabolite of dietary fiber. Some of these fatty acids bind to G-protein-coupled receptor (GPCR) including GPR41 (Ffar3) and GPR43 (Ffar2) (12, 13), playing a protective role in immune-mediated diseases partially through enhancing the differentiation of regulatory T cells (Treg) in the intestine and enhancing the integrity of the intestinal epithelial barrier. During intestinal inflammation, SCFAs activate GPR43 on neutrophils and enhance the expression of L-selectin, which is a key molecule for leukocyte rolling on vascular endothelium, leading to the stimulation of migration, production of reactive oxygen species, and enhancement of phagocytosis (14). In chronic murine colitis mice, Gpr43 KO animals showed diminished intestinal migration of polymorphonuclear leukocytes (PMNs) (15). In acute inflammation induced by LPS, the intravascular rolling velocity of GPR43-deficient neutrophils was reduced significantly, and increased numbers of neutrophils were found in the lamina propria (16). In patients with CD, the infiltration of GPR43+ PMN infiltration was more severe in active CD patients who consumed enteral nutrition with dietary fiber, suggesting that the use of dietary fiber in enteral nutrition by active CD patients might increase GPR43+ PMNs infiltration in the colon mucosa (17). Recently, SCFAs, which are released by *Fusobacterium nucleatum*, trigger chemotaxis of human neutrophils (18). Butyrate downregulates the differentiation of DCs with reduced expressions of CD80, CD83, and MHC II and promotes IL-10 production (19), suggesting that butyrate can interfere the maturation of DCs and antigen-specific T-cell proliferation and suppress the intestinal inflammation. ILCs also express SCFA-sensing receptors, and GPCR triggered by SCFAs promotes ILC proliferation by co-stimulating the activation of phosphoinositide 3-kinase, Stat3, Stat5, and mammalian target of rapamycin (20). Ffar2 signaling by SCFAs also activates AKT and STAT3 signaling and increases ILC3-derived IL-22 (21), which is essential for epithelial barrier function in early phase upon inflammatory damage, while in chronic phase, CD4+ cells are the main source of IL-22 in the lamina propria. Therefore, SCFAs play a pivotal role for shaping ILC3-mediated gut immunity and adaptive immunity. Supplementation of butyrate-producing gut symbiont in human has been regarded as valuable intervention for several gut-related diseases (22). Meanwhile, butyrate might be partly responsible for inducing eosinophilic esophagitis *via* IL-4/Ffar3/Th2 cytokines pathway (23). A recent study showed that the esophagus is colonized with SCFA-producing bacteria (24), and butyrate enhances expression of Ffar3 on Th2, leading to increased secretion of IL-5 and accumulation of eosinophils in the tissue (23). The Ffar3-SCFA axis in allergic inflammation needs to be further investigated.

#### Succinate

Succinate is an intermediate of the tricarboxylic acid cycle in the mitochondria. Succinate is accumulated at a higher concentration in the inflamed mucosa of IBD patients compared with control patients (25), and its signaling through succinate receptor (GPR91 or SUCNR1) modulates immune function. Although the distal GI tract contains a large amount of microbes that produce succinate as a byproduct of anaerobic fermentation (26), the major provider of circulating succinate is supposed to be the intestinal tissue itself (27). In monocyte-derived DCs, SUCNR1 stimulated by succinate induces intracellular calcium mobilization and ERK1/2 phosphorylation (28), promoting the migration and production of inflammatory cytokines. Succinate also enhances the antigen-specific activation of T cells *via* SUCNR1 on DCs (28). In a diarrheal animal model, accumulated luminal succinate regulates transepithelial Cl^−^ secretion in epithelial cells and increases colonic fluid secretion with enhanced colonic inflammatory responses *via* activation of MyD88-dependent TLR4 in macrophages (29). Mast cell also expresses SUCNR1 and regulates homeostatic intestinal barrier function by a chymase/Mcpt4-dependent mechanism (30). During helminth or protist infection, tuft cells express IL-25 and activate ILC2s *via* the IL-17RB receptor (IL-25R), leading to the production of IL-13, which promotes the differentiation of tuft and goblet cell (31). This tuft-ILC2 circuit is positively enhanced by succinate. Tuft cells sense succinate *via* GPR91 to trigger the IL-25–ILC2–IL-13-dependent immune circuit and intestinal remodeling (32, 33). Activated circuit leads to crypt fission and lengthening of the intestine and enhances resistance to new helminth infection. It is recently reported that the IL-25-dependent tuft cell circuit requires macrophage migration inhibitory factor (MIF) (34), which is constitutively expressed by epithelial cells and immune cells. The expression of CXCR4, an MIF receptor, on both ILC2s and macrophages indicates that MIF-induced migration of these cells may be essential for the activation of the IL-25-dependent tuft cell circuit (34). In food allergy, tuft cells are supposed to initiate type 2 immunity in response to increased luminal succinate (35), leading to recruitment of ILC2s and mast cells and secretory-antigen passages in the small intestine.

### Aryl hydrocarbon receptor ligand, tryptophan metabolites

Tryptophan, a kind of essential amino acid, is metabolized *via* three pathways into indole and its derivatives: the serotonin pathway in enterochromaffin cells, the kynurenine pathway in epithelial and immune cells, and the indole pathway in the gut microbiota (11). Among them, kynurenine and indole metabolites are ligands of aryl hydrocarbon receptor (Ahr) (11). Ahr responds to cellular and dietary ligands and regulates the development and function of both innate and adaptive immune cells (36). Gut-associated Treg cells highly express Ahr compared with those of Tregs in other organs such as the spleen and might exert suppressive role for intestinal immune homeostasis (36). Tryptophan derivatives also activate Ahr/IL-22 pathway in ILC3 (RORγt^+^ ILC) (37). CCR6, a target gene of RORγt, is involved in the migration of cells *via* CCL20 under physiological and inflammatory conditions, which play an important role in adaptive immunity (38). Considering that Ahr deficiency decreases the expression of CCR6, tryptophan might be able to enhance migration of ILC3 *via* Ahr modulation of CCR6 (39). Furthermore, there is an Ahr-mediated crosstalk between T cells and ILC3s. ILC3s inhibit Th17-mediated intestinal inflammation through Ahr signaling, suggesting that the balance between ILC3s and Th17 cells are regulated by Ahr (40). Ahr might be a potential therapeutic target in food allergy. Indole-3-carbinole (I3C) supplementation, which is abundant in cruciferous plants and a kind of Ahr ligand, attenuates symptoms of peanut allergy in mice *via* stimulation of Ahr expressed on DCs and the intestinal epithelium (41). In addition, retinaldehyde dehydrogenase (RALDH) expressed in DCs are activated *via* stimulation of Ahr by I3C (41), leading to the generation of retinoic acid and subsequent regulatory T cells. In IBS and CD, the activity of indoleamine-2.3-dioxygenase (IDO), one of key primary kynurenine pathway enzymes, has been reported to be increased along with elevated serum kynurenine/tryptophan ratio (42, 43), suggesting that modulation of tryptophan metabolites could be a useful therapeutic strategy.

### Fat

Fat is important for the structure of cellular lipid membranes and is an essential source of energy. On the other hand, fat alters barrier structure and the gut microbiota by inducing enhanced intestinal permeability (44), and dietary intake of total fat has been shown to be related with the incidence of CD (45). Several reports showed that dietary fat modulates migration of immune cells. Dietary fat enhances homing of circulating lymphocytes through an interaction between fat-induced adhesion molecules on lymphocytes such as α4β7 integrin and counter-receptors on endothelial cells such as MAdCAM-1 (46). In the intestine, NKp46+ ILC3s mainly producing protective cytokine IL-22 are decreased through dysbiosis induced by a high fat diet, resulting in loss of enterocyte proliferation and subsequently low-grade intestinal inflammation (47). Trans fatty acids contain trans carbon–carbon double bonds and are mainly produced in the food production process. Trans fatty acids act on macrophages in the lamina propria of the small intestine and liver *via* fatty acid transporter (CD36) to stimulate the secretion of IL-12, which promotes the secretion of tumor necrosis factor alpha (TNF-α) from ILC1 and causes a stronger inflammatory response by changing RORγt-positive ILC3 to T-bet-positive ILC3 with ILC1-like effects (48). Exposure of the intestines to dietary fat may contribute to food allergy through inducing mast cell accumulation and releasing mast cell protease 1 (MCP1), leading to the increased intestinal permeability and the promotion of passage of allergens through the epithelium (49).

### Vitamins A and D

Vitamin A is found in many types of food as all-trans-retinol or β-carotene. In the tissues, retinol and carotene are oxidized to retinal by alcohol dehydrogenases (ADHs), and retinal is then oxidized to the active metabolite retinoic acid (RA) by RALDH. RALDH is detected in some gut-associated cells such as intestinal epithelial cells (IECs) and DCs in Peyer’s patches (PPs) and MLNs. Vitamin A absorbed from the small intestines is processed to retinol and then to RA (50). These retinoids physiologically exist at high concentrations in the gut. Vitamin D is synthesized in the skin under sunlight exposure and is provided from the diet. RA exerts strong immunological effects on both innate and adaptive immune response (51). RA plays important roles in the differentiation and function of T and B cells and influence their homing properties. The induction of Treg is enhanced by RA and TGFβ (52). Meanwhile, vitamin D, in its hormonally active form of 1,25-dihydroxyvitamin D3 (1,25D3), has an immunosuppressive effect by inhibiting the proliferation, the expression of homing receptors, the secretion of cytokines like IL-2 and interferon gamma (IFN-γ) in T cells, and CD8 T-cell-mediated cytotoxicity (53). The role of RA in controlling intestinal ILC has also been revealed. Reduced RA leads to impaired intestinal immunity due to a lack of IL-22 production from ILC3 (54). RA plays another role in controlling the migration of ILC1 and ILC3 into the intestinal tissue by upregulating the trafficking receptor CCR9 and α4β7 (55). On the contrary, enhanced cytokine production and expression of integrin α4β7 on ILCs by RA + IL-2 are suppressed by the addition of 1,25D3 by at least 30% (56). Considering that low vitamin D is related to the development of food allergy (57), the mechanism of which is involved with ILCs (58), the balance between vitamins A and D might be an important factor in the ILCs-related diseases like IBD. Meanwhile, gut-dysbiosis-induced dysregulated bile acids (BAs) induce enhancement of RA signaling in mucosal DCs, leading to food allergen-specific IgE and IgG1 (59), implying that BA-RA signaling may change Th2 cells prime DCs.

### Lipid mediators

Lipid mediators can be classified into three categories (60): class 1, arachidonic acid (AA)-derived eicosanoids including prostaglandins (PGs), leukotrienes (LTs), and their relatives; class 2: lysophospholipids or their derivatives such as platelet-activating factor (PAF), lysophosphatidic acid (LPA), and sphingosine-1- phosphate (S1P), which possess either glycerol or sphingosine backbone; and class 3, anti-inflammatory lipid mediators derived from ω-3 polyunsaturated fatty acids (PUFAs) such as eicosapentaenoic acid (EPA) and docosahexaenoic acid (DHA). Immunologically active lipid mediators are mainly derived from AA. Representative lipid mediators are reviewed in this section.

#### Prostaglandins

The most dominant bioactive prostaglandins include PGD2, PGE2, and PGI2. Among them, PGE2 is an important lipid mediator in both acute and chronic inflammations, regulating the activation, maturation, migration, and cytokine production of several immune cells including innate immunity such as macrophages, DCs, neutrophils, and natural killer (NK) cells through binding to the PGE2 receptors 1–4 (EP1–4) (61). PGE2 is generated from arachidonic acid derived from membrane phospholipid catalyzed by phospholipase A2 under inflammation (62). Bacterial pathogens and their structural component LPS promote PGE2 synthesis by neutrophils during infections (63), which inhibits activation and aggregation of neutrophils *via* EP2 stimulation, leading to the suppression of formylmethionyl-leucyl-phenylalanine/phospholipase D pathway (64). EP4 receptor stimulation also downregulates eosinophil function (65), implying the therapeutic potential of EP4 agonists against eosinophilic diseases. In addition, PGE2 is supposed to decreases basophil activation in patients with food-induced anaphylaxis *via* EP4 (66). Upon stimulation of PGE2 on macrophages, NAPDH oxidase and nitric oxide radicals inside the cell are inhibited and suppress the activity of macrophages (67). PGE2-EP4 signaling promotes the homeostasis of ILC3 and suppresses systemic inflammation through ILC3/IL-22 axis (68). PGD2, mainly synthesized in mast cells and tuft cells, exerts its biological actions through the chemoattractant receptor-homologous molecule expressed on Th2 cells (CRTH2) expressed on several immune cells including DCs, mast cells, eosinophils, and ILCs (69). Although the role of PGD2 signaling in allergic response is still controversial (70), some recent studies highlighted an anti-inflammatory role of PGD2 derived from mast cell in food allergic responses by inhibiting mast cell chemoattractants (70) and a protective role of PDG2 derived from tuft cell during helminth infection *via* negative regulation of Th2-related pathway (71). PGD2/CRTH2 induces migration of ILC2 and secretion of cytokines including IL-13 and upregulates IL-33/IL-25 receptors, leading to allergic inflammatory responses (72). Endothelial cells are involved in the migration of neutrophils by producing PGD2 *via* D prostanoid1, another PGD2 receptor on neutrophils (73). PGI2 is highly expressed in the lung tissue, and its signaling has the capacity to attenuate allergic airway inflammation by suppressing ILC2 cytokine secretion and proliferation (74). Recently, a reduction in intestinal PGI2 in IBD patients and its protective role in colitis by enhancing intestinal epithelial barrier integrity has been reported (75), suggesting the therapeutic role by supplementation of PGI2.

#### Sphingosine-1-phosphate

Sphingosine-1-phosphate (S1P), a biologically active sphingolipid regulating trafficking and activation of immune cells, is rich in the blood and lymph (76). S1P is derived from the cell membranes from sphingomyelin and is produced mainly by erythrocytes, endothelial cells, and platelets, and degraded by S1P lyase in the lymphoid tissues. S1P gradient is established by this metabolic pathway between the blood/lymph and lymphoid tissues, which allows trafficking of immune cells including lymphocytes and DCs (77). S1P gradient is also involved with migration of ILCs. IL-25-induced activation of intestinal ILC2s downregulates CD69 expression and upregulates S1P receptors, moving across the villus lymphatic endothelium in an S1P-depndent manner (78). The drug FTY720, fingolimod, the first S1P modulator, blocks egress of the lamina propria ILC2s into the central lymphatic and prevents accumulation in distal organs including the lung (79). S1P receptor is also expressed on human ILC1 and ILC3s on the cell surface, and S1P modulator reduces the number of lamina propria-resident ILC3s (80).

#### ω-3 PUFAs

Omega-3 polyunsaturated fatty acids (ω-3 PUFAs) are essential fatty acids obtained from diet and have several potential benefits for human health. ω-3 PUFAs modulate the gut microbiota directly or indirectly and influence the function of white adipose tissue, contributing to the occurrence and development of several diseases including obesity and non-alcoholic fatty liver disease (81). ω-3 PUFAs reduce inflammation *via* three main pathways: 1) suppressing the activation of proinflammatory MAPK and nuclear factor kappa B (NF-κB) signaling pathway, 2) reducing inflammatory precursor substance including endotoxin and cyclooxygenase 2, and 3) altering the regulation of the expression of inflammation-related genes (81). In macrophages, DHA or EPA downregulates LPS-induced increase in cytokine gene expressions (82). DHA metabolite resolvin D1 reduces neutrophil migration *via* a decrease in actin polymerization (82). The effect of PUFAs on ILCs has not been clarified yet. Clinically, a meta-analysis investigating the long-term effects of PUFAs on IBD showed that supplementations with PUFAs have almost no effect on treatment of IBD (83).

### Neuropeptides

The intestine contains the largest amount of lymphoid tissue in the body, and a neural network, as many neurons as the spinal cord, ensures intestinal homeostasis and function (84). Intestinal neurons are classified into intrinsic nervous system (enteric nervous system; ENS) and extrinsic nervous system (sympathetic and parasympathetic autonomic nervous system) (84). Parasympathetic neurons (vagal nerve) of the digestive tract interfaces with ENS. ENS contains myenteric (of Auerbach) plexus and inner submucosal (or Meissner) plexus (84). To manage threats to the gut, inflammation is finely tuned partly by the nervous system, which is appropriate for tissue defense. Immune cells receive neuronal signals by placing them near the neuron and expressing receptors for neuronal-cell-derived molecules, and, reciprocally, neurons receive immune-derived cytokines and neurotransmitters, which can modulate neuronal function. Neuro-immune interactions are multilaterally involved in tissue physiology and several disease conditions including chronic inflammatory disorders (85).

Mast cells are often localized close to nerve endings, a key position to act as the intermediate cells between the nervous and immune system. Histamine, serotonin, and tryptase are mast cell mediators and induce nociceptor sensitization (sensory neuron), leading to the release of neuropeptides from nociceptor such as calcitonin gene-related peptide (CGRP), corticotropin-releasing hormone (CRH), vasoactive intestinal peptide (VIP), neuromedin U (NMU), and substance P (86). Activated mast cells by neuropeptides degranulate and release cell mediators, thus inducing a bidirectional positive-signaling loop and subsequent neurogenic inflammation (86). Neuro-mast cell interactions play an important role in intestinal disorders including IBD, irritable bowel syndrome, and food allergy (86). In addition to sensory neurons, mast cell function is controlled by sympathetic and parasympathetic neurons. Mast cells are inhibited to release histamine *via* stimulation of β2-adrenergic receptor (87). Vagal nerve endings also communicate with mast cells and induce trophic effects (88). Furthermore, acetylcholine (Ach) released from vagus nerve is involved with the “cholinergic anti-inflammatory pathway”, a key mechanism which is mainly stimulated via α7 nicotinic Ach receptor expressed on muscularis resident macrophages (89).

The sympathetic nervous system through adrenergic receptor signaling modulates the function of DCs, including migration, antigen cross-presentation, and cytokine production. Signaling through β2-adrenergic receptor on DCs inhibits antigen cross-presentation to CD8 T cells and suppresses production of cytokines including TNF-α and IL-6 by suppressing NF-κB and JNK signaling (90). In contrast, the stimulation of α1-adrenergic receptors enhances antigen uptake and increases DC migration to draining lymph nodes (91).

Substance P (SP) exerts a wide range effect, and the most widely known roles are in nociception and neurogenic inflammation primarily through the neurokinin-1 (NK-1) receptor. In ulcerative colitis, rectal SP is increased and correlated with disease activity (92). SP is strongly involved in proinflammatory roles of immune cells including innate immunity (92). Adhesion molecules including intercellular adhesion molecule-1 and endothelial leukocyte adhesion molecule-1 are induced by SP, along with increased chemokines, such as macrophage inflammatory proteins, monocyte chemoattractant protein-1, CCL5, and IL-8. In addition, SP elicits local vasodilatation and induces vascular hyperpermeability, thus ensuring extravasation, migration, and accumulation of immune cells at the inflammatory lesion.

VIP is the most abundant gut neuropeptide. VIP-producing neurons (VIPergic neurons) have been detected in various lymphoid organs including PPs (93). VIP can modulate the transendothelial migration of T cells through the post-capillary venule of ileal PPs and inhibit migration into the interfollicular lymphatics, possibly by elevation of the intracellular cAMP concentrations (94). VIPergic neurons in the lamina propria are located close to ILC3s that selectively express VIP receptor type 2 (VIPR2). Activated VIPergic neurons by food consumption antagonize production of IL-22 by ILC3 *via* VIPR2, while VIP increases lipid absorption (95).

Neuromedin U receptor 1 (Nmur1) was identified by genome-wide transcriptional profiling of ILC2 by comparing with T helper cells and other innate lymphoid cells (96). Neuromedin U (NMU) is a fast and strong positive regulator of ILC2 (97). ILC2s selectively express Nmur1, and mucosal neurons express NMU, which is expressed in cholinergic neurons in the small intestine and upregulated after helminth infection. NMU directly induces ILC2 proliferation and production of IL-5, IL-9, and IL-13, and amphiregulin to protect against helminth infection. Nmur 1 is also expressed on other type 2 immune cells including Th2 and eosinophils, and NMU elicits migration of type 2 immune cells (98). Mucosal neurons are located adjacent to ILC2s, and these neurons directly sensed worm products and alarmins to induce NMU, leading to the production of innate type 2 cytokines (97), while CGRP antagonizes many actions of NMU and IL-33 but promote IL-5 production (99). Neuron-ILC2 cell units work immediately for tissue protection through coordinated neuroimmune sensory responses (96).

The receptors of CRH, CRHR1 and CRHR2, have been detected throughout the gastrointestinal tract in various cell types including enterochromaffin cells, neuronal cells, and immune cells (eosinophils, mast cells, and T-helper lymphocytes in the lamina propria) (100). CRH plays a crucial role in the stress-induced pathophysiology of IBS. Psychological stress induces intestinal permeability *via* CRH-mediated mast cell activation (101), and CRH has anti-inflammatory effects in TLR4-dependent innate-immunity-related colitis (102), suggesting that CRH might be a therapeutic target in IBD.

## Conclusion

Dietary nutrients are metabolized to form important modulators of innate immunity and adaptive immunity. Since overactivation of innate cells including ILCs have been implicated to contribute to immune pathology including IBD and food allergy, a better understanding of these complex network may bring a new therapeutic target in the future.

## Author contributions

MH, SM, and RH drafted the manuscript. All authors contributed to the article and approved the submitted version
